# National trends in cardiovascular health metrics among Iranian adults using results of three cross-sectional STEPwise approaches to surveillance surveys

**DOI:** 10.1038/s41598-020-79322-x

**Published:** 2021-01-08

**Authors:** Fatemeh Rahmani, Samaneh Asgari, Davood Khalili, Ali Siamak Habibi Moeini, Maryam Tohidi, Fereidoun Azizi, Farzad Hadaegh

**Affiliations:** 1 Prevention of Metabolic Disorders Research Center, Research Institute for Endocrine Sciences, Shahid Beheshti University of Medical Sciences, Tehran, Iran; 2 Department of Biostatistics and Epidemiology, Research Institute for Endocrine Sciences, Shahid Beheshti University of Medical Sciences, Tehran, Iran; 3 Endocrine Research Center, Research Institute for Endocrine Sciences, Shahid Beheshti University of Medical Sciences, Tehran, Iran

**Keywords:** Cardiology, Health care

## Abstract

To examine the trends of 7 cardiovascular health metrics (CVH metrics) incorporate of smoking, physical activity, diet, body mass index (BMI), fasting plasma glucose (FPG), total cholesterol (TC), and blood pressure (BP) level during three cross-sectional STEPwise approaches to surveillance (STEPS), 2007–2016, among Iranian adults. The study population consisted of 19,841 women and 17,243 men, aged 20–65 years. The CVH metrics were categorized as ‘ideal’, ‘intermediate’, and ‘poor’. The sex-stratified weighted prevalence rate of each CVH metrics was reported. The conditional probability of each poor versus combined intermediate and ideal metric was analyzed using logistic regression. In 2016 compared to 2007, the prevalence of poor BP level (20.4% vs. 23.7%), smoking (13.7% vs. 23.8%), TC ≥ 240 mg/dl (2.4% vs. 11.2%) and FPG < 100 mg/dl (75.6% vs. 82.3%) declined, whereas poor physical activity level (49.7% vs. 30%), poor healthy diet score (38.1% vs. 4.1%), BMI levels ≥ 25 kg/m^2^ (62.8% vs. 57.8%) increased. Despite a high prevalence of obesity among women, it remained constant in women but showed an increasing trend in men; moreover, the trends of low physical activity and current smoking were better for women. Despite some improvement in CVH metrics, < 4% of Iranian adults meet ≥ 6 CVH metrics in 2016; this issue needs intervention at the public health level using a multi-component strategy.

## Introduction

Cardiovascular Disease (CVD) persists as a critical public health problem despite the great global effort to reduce cardiovascular risk factors through primary prevention^1^. Smoking, physical inactivity, high blood pressure, high cholesterol level, unhealthy diet alongside obesity, and glucose intolerance status contribute to persistent CVD. Achieving favorable changes in healthy lifestyle factors (even slightly) can have a considerable effect on decreasing the epidemiologic and economic health burden^2^.

Based on evidence from randomized clinical trials, the American Heart Association (AHA) established the 2020 strategic impact goals, which incorporated 7 cardiovascular health metrics (CVH metrics), consisting of four health behaviors [body mass index (BMI), smoking status, physical activity, and diet] and three health factors [blood pressure (BP), fasting plasma glucose (FPG), and total serum cholesterol (TC)] for improvement of cardiovascular health^3^. There is convincing evidence that a more ideal CVH metrics is accompanied by reduced risk of CVD and non-CVD outcomes such as cancer, depression, cognitive impairment, and diabetes^4,5^.

Several cross-sectional studies have focused on the prevalence of CVH metrics in different populations. The distribution of ideal CVH metrics (characterized by achieving 6 or 7 ideal CVH metrics) between 2010 and 2015 was reported to be as low as 0.5–12% in the United States (US) and 0.3–10% in the non-US population^5^. Among the seven CVH metrics, the highest and lowest prevalence for ideal CVH metrics was attributed to smoking status and diet, respectively^5^. However, survey data showing the trends of CVH metrics among the general population are very limited. To the best of our knowledge, few population-based studies have been conducted among US, Canadian and Korean populations that examined the trends of all 7 ideal cardiovascular health metrics. Accordingly, two American National Health and Nutrition Examination Surveys (NHANES) between 1998 and 2010, the Korean National Health and Nutrition Examination Survey (KNHANES) between 2005 and 2009, and a Canadian study carried out from 2003 to 2011 showed no improvement in the prevalence of ideal CVH metrics^2,4,6,7^.

The Middle East and North Africa (MENA) region is considered to carry a high absolute burden of CVD. Risk factors such as rapid urbanization, changing lifestyle including physical inactivity and nutritional transition to high caloric intake, political instability, social conflict, terrorism, and lack of comprehensive preventive programs over the past decades are the main problems in this region^8^. Ischemic heart disease, the most important cause of death in Iran in the year 2016, was responsible for 40% and 44% of all deaths in men and women, respectively^9^. Our previous cross-sectional study among Tehranian residents showed a very low prevalence of ideal CVH metrics in 1999–2011^10^.

To the best of our knowledge, this is the first nationwide gender-specific report on the trend of CVH metrics in the MENA region, using the AHA’s 2020 impact goals among Iranian adults in the scope of the STEPwise approach to surveillance (STEPS) during 2007–2016.

## Materials and methods

### STEPS 2007–2016

This study is secondary data analysis on the World Health Organization (WHO) STEPS surveys. Data was collected using a random multistage cluster sampling method in non-hospitalized and non-institutionalized Iranian adults, by trained interviewers during a household meeting. The STEPS survey was conducted in seven stages, between 2004 and 2016, four of which included laboratory measurements on a subsample population. In the current study, we used three STEPS, 2007, 2011, and 2016; which had similar methods for the measurement of all needed CVH metrics variables. Despite slight differences in sampling designs between the 3 surveys, and the smaller sample size in 2011 (due to financial constraints and economic problems), samples were representative of the Iranian adult population in all 3 surveys. Further details regarding STEPS have been previously published^11–13^. We confirm that all research was performed following relevant guidelines, and confirming that informed verbal consent was obtained from all participants and/or their legal guardians. This study was approved by the Institutional Review Board (IRB) of the Research Institute for Endocrine Sciences (RIES), Shahid Beheshti University of Medical Sciences, Tehran, Iran, and all participants provided written informed consent.

### Study population

**Figure 1:**
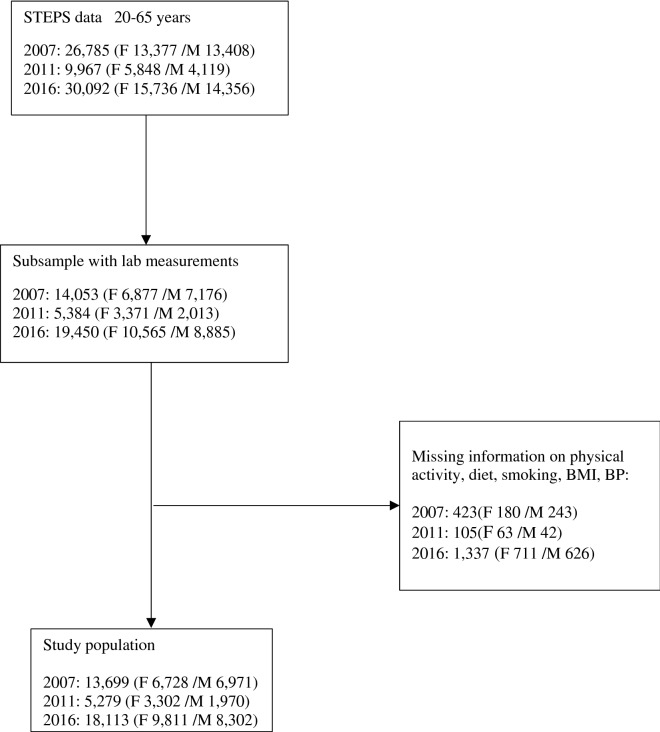
Flow chart of the study population in STEPS 2007–2016.

For trends in CVH metrics, we used data from STEPS 2007 (n = 26,785), 2011 (n = 9967), and 2016 (n = 30,092). FPG and TC values were available for a subsample of adults aged 20–65 years [(2007 = 14,053), (2011 = 5384), (2016 = 19,450)]. After removing subjects with missing data on all 7 CVH metrics, 37,091 adults [(2007 = 13,699), (2011 = 5279), (2016 = 18,113)] remained for data analysis (Fig. 1).

### Medical history, clinical examination, and laboratory measurements

Demographic information on sex, age, education, occupation as well as cigarette smoking, physical activity, and drug consumption were obtained from participants using a standard questionnaire based on the STEPS survey^14^. Systolic and diastolic blood pressure (SBP and DBP) were recorded after at least 10 min of rest, three times within 5 min of each other, in a seated position from the right arm, using an appropriately-sized standard cuff sphygmomanometer (Omron M7 in 2007 and 2011, Beurer in 2016). The mean value was considered as the subject’s SBP and DBP. Height was measured with a portable, inflexible measuring bar in standing position with no shoes on, with a precision of 0.1 cm. Weight was measured using a digital scale in light clothing without shoes on and recorded to the nearest 0.1 kg. BMI was calculated as weight in kilograms divided by height in meters squared. For biochemical measurements, according to the standard protocol, 10–12 ml of the venous blood sample was drawn from each participant after at least 8 h of overnight fasting. Samples were sent to collaborating centers in 2007 and 2011, but in 2016 samples were sent to the Central Reference Laboratory of the Ministry of Health of Iran (Tehran, Iran) under cold chain conditions(4–8 °C). All samples were promptly centrifuged (1500 rpm for 10 min at standard room temperature: 21 °C). FPG was measured via enzymatic colorimetric method using glucose oxidize and TC level was checked via enzymatic photometric method using cholesterol esterase and cholesterol oxidase in 2007 and 2011, whereas the auto analyzer (Cobas C311 Hitachi High-Technologies Corporation. Tokyo Made in Japan) was used in 2016.

### Definition of CVH metrics

We used AHA’s 2020 Impact Goals which defined three CVH metrics categories; ‘ideal’, ‘intermediate’, and ‘poor’(Table1)^16^. The subgroup of smoking status consists of current, former, and never-smokers, which were attributed ‘poor’, ‘intermediate’, and ‘ideal’ status, respectively. The AHA’s definition of ‘ideal’ smoking status includes never or quit smoking > 12 months, ‘intermediate’ groups include quit smoking less than 12 months ago, and ‘poor’ groups include smoking daily or occasionally. In the questionnaire, we categorized the ‘current’ smoker as a participant who smoked daily or occasionally, any amount of all kinds of tobacco (cigarette, pipe, hookah, etc.) at the time of the interview and ‘former’ as someone who had a history of smoking but had since quit. According to our questionnaire, we used average metabolic equivalent tasks score (METs) to define physical activity instead of using the duration of physical activity (used in AHA’s 2020 impact goals), which were calculated based on the global physical activity questionnaire (GPAQ). The GPAQ is a standardized questionnaire of physical activity developed by the WHO^15^. ‘ideal’, ‘intermediate’, and ‘poor’ physical activity was defined as physical activity of ≥ 1500 MET mins/wk, 600–1500 MET mins/wk, and < 600 MET mins/wk, respectively. The corresponding metrics for BMI were ‘ideal’ (< 25.0 kg/m^2^), ‘intermediate’ (25.0–29.9 kg/m^2^) and ‘poor’ (≥ 30.0 kg/m^2^). AHA’s healthy diet score was defined by the sum of the following 5 components, allocating 1 point each for the consumption of fruits and vegetables (≥ 4.5 cups/d), fish (≥ two 3.5-oz servings/wk), fiber-rich whole grains (≥ three 1-oz equivalent servings/d), sodium(< 1500 mg/d), and sugar-sweetened beverages(< 36 oz./wk)^16^. Considering the limited data in our questionnaire, the modified healthy diet score was used based on only two components, daily fruits and vegetables, and fish servings in all 3 surveys. Participants were placed in the ’ideal’ group if their fruit and vegetable consumption was ≥ 4 cups/d, and consumption of fish ≥ two 3.5-oz servings/wk. The ‘poor’ group comprised participants who consumed less than 2 cups/d fruits and vegetables, and without consumption of fish. All other participants were categorized as the ‘intermediate’ group. Statin therapy for high cholesterol level was recorded only for STEPS 2016, therefore we modified AHA’s definition of TC status, without considering treatment information in 2007 and 2011. We categorized participants as ‘ideal’ if TC < 200 mg/dl, ‘intermediate’ if TC level was 200–239 mg/dl, and ‘poor’ if they had a TC level equal or greater than 240 mg/dl. According to FPG level, participants were ranked as ‘ideal’ if untreated FPG was lower than 100 mg/dl, ‘intermediate’ if untreated FPG was 100–125 mg/dl or treated to lower than 125 mg/dl, and ‘poor’ if they had an FPG level equal or greater than 126 mg/dl. Subgroup classification of blood pressure level consisted of ‘ideal’ (SBP < 120 mmHg/DBP < 80 mmHg, untreated), ‘intermediate’ (120 mmHg ≤ SBP ≤ 139 mmHg/80 ≤ DBP ≤ 89 mmHg, or treated to ≤ 139/89 mmHg), and ‘poor’ (SBP ≥ 140 mmHg/DBP ≥ 90 mmHg). To calculate the total CVH metrics score, in the first step we recoded each metric as a binary variable, assigning a score of 1 point to the ‘intermediate’ or ‘ideal’ category versus 0 points for the ‘poor’ category. A CVH metric total score was attained by summing up the newly defined variables. A total CVH metric score was categorized as ‘‘ideal’ if the total CVH metric score was 6–7, ‘intermediate’ if the score was 3–5, and ‘poor’ if the CVH metric score was less than 2.

**Table 1:** The modified American Heart Association 2020 impact goals of ideal, intermediate, and poor CVH metrics.

CVH metrics	Ideal	Intermediate	Poor
Current smoking	Never	Former	Yes
Physical activity level	≥ 1500 METs min/week	600–1500 METs min/week	< 600 METs min/week
BMI, kg/m^2^	< 25.0	25.0–29.9	> 30.0
Healthy diet score	F&V (≥ 4 cups/d) and fish (≥ two 3.5-oz servings/week)	^a^Not included in ideal or poor group	F&V (< 2 cups/d), no fish consumption
TC, mg/dL	< 200	200–239	≥ 240
FPG, mg/dL	< 100, untreated	100–125 or treated	≥ 126
SBP/DBP, mmHg	< 120/80, untreated	120–139/80–89, or treated	≥ 140/90

CVH metrics, cardiovascular health index; METs, metabolic equivalent tasks; BMI, body mass index; F&V, fruit and vegetable; TC, total cholesterol; FPG, fasting plasma glucose; SBP, systolic blood pressure; DBP, diastolic blood pressure.

^a^F&V (≥ 4 cups/d) without fish, F&V(2–4 cups/d) and fish (≥ one 3.5-oz serving/week), F&V (< 2 cups/d) and fish ((≥ one 3.5-oz serving/week), fish (≥ one 3.5-oz serving/week) without F&V.

### Statistical analysis

In the current study, we used STEPs with a complex sampling design. To achieve a representative estimation of the Iranian population, a survey analysis was performed and weights were generated using categories of age, sex, area of residence (rural/urban), and 30 provinces, according to the national population of Iran (census, 2011). All data from 2007, 2011, and 2016 STEPs were pooled. The weighted prevalence rates (95% CI) of each health metric were estimated using Taylor-linearized variance estimation. The conditional probability of each poor vs. combined intermediate and ideal metric was analyzed by logistic regression, wherein the STEPs cycles were considered a continuous independent variable, and linear trends in the probability of each health metric were examined. Differences across the sexes were examined by using interaction terms (e.g., sex × STEPs cycle). All analyses were performed using STATA 14, and *p* value < 0.05 was considered statistically significant.

## Results

Demographic characteristics of the study participants are described in Table 2. The mean CVH metrics score increased slightly during 2007–2016 (4.7 in 2007 [95% CI 4.7–4.8]; 4.9 in 2011 [95% CI 4.8–5.0]; 5.0 in 2016 [95% CI 4.9–5.0]).

**Table 2:** Demographic characteristics of the Iranian adult population in STEPS 2007–2016.

	2007			2011			2016		
	Male	Female	Total	Male	Female	Total	Male	Female	Total
No	6971	6728	13,699	1970	3302	5272	8302	9811	18,113
Mean age (year)	41.3 (41.1–41.6)	41.0 (40.8–41.3)	41.2 (41.0–41.3)	40.7 (40.3–41.2)	40.8 (40.2–41.4)	40.8 (40.4–41.1)	41.7 (41.5–41.9)	42.0 (41.8–42.3)	41.8 (41.7–42.0)
Urban% (95% CI)	67.9 (66.8–68.9)	69.6 (68.6–70.6)	68.6 (67.9–69.3)	78.7 (76.7–80.6)	74.0 (72.5–75.5)	76.0 (74.8–77.2)	77.1 (76.5–77.7)	75.8 (75.2–76.3)	76.4 (76.0–76.9)
Antihypertensive medication% (95% CI)	4.0 (3.5–4.6)	7.5 (6.9–8.1)	5.5 (5.1–6.0)	4.8 (3.9–5.8)	8.4 (7.5–9.5)	6.9 (6.2–7.7)	12.1 (11.3–13.0)	15.6 (14.8–16.3)	13.9 (13.3–14.4)
Anthyperglycemic medication% (95% CI)	2.9 (2.4–3.4)	4.6 (4.1–5.2)	3.6 (3.3–4.0)	4.0 (3.2–5.0)	5.7 (4.9–6.7)	5.0 (4.4–5.7)	3.5 (3.1–4.0)	4.9 (4.4–5.3)	4.1 (3.9–4.5)
Stain treatment% (95%CI)	N/A	N/A	N/A	N/A	N/A	N/A	5.0 (4.5–5.4)	6.3 (5.9–6.7)	5.6 (5.3–5.9)

STEPS, STEPwise approach to surveillance.

As shown in Table 3, in 2016 compared to 2007, the prevalence of current smoking (13.7% vs. 23.8%), poor TC (2.4% vs. 11.2%), and poor BP level (20.4% vs. 23.7%) declined, whereas poor physical activity level (49.7% vs. 30.0%), poor healthy diet score (38.1% vs. 4.1%) as well as poor and intermediate BMI levels (23.7% vs. 21.6% and 39.1% vs. 36.2%, respectively) were significantly increased. Regarding poor FPG level, we did not find a consistent change in prevalence during these years, considering significant overlap features in their confidence intervals, but the prevalence of ideal glucose level (FPG < 100 mg/dl) decreased [82.3% in 2007 (95% CI 81.5–83.1%) to 75.6% in 2016 (95% CI 74.8–76.3%)]. The prevalence of all 7 ideal CVH metrics was less than 0.5% during this period. The prevalence of meeting 6 CVH metrics was 5.5% in 2007, 6.7% in 2011, and 3.4% in 2016. Among different CVH metrics, those with 3 and 4 indices had the highest prevalence in each of the three STEPS surveys, and the prevalence of CVH metrics in these groups did not change significantly during 2007–2016.

**Table 3:** Prevalence of cardiovascular health metrics in adults in STEPS 2007, 2011, 2016.

	2007		2011		2016	
	No	Prevalence (95%CI)	No	Prevalence (95%CI)	No	Prevalence (95%CI)
**Smoking status**						
Never	9822	67.8 (67.0–68.8)	4417	83.2 (81.9–84.7)	14,197	79.6 (78.8–80.3)
Former	1072	8.4 (7.7–9.0)	209	3.5 (2.8–4.1)	1436	6.7 (6.3–7.2)
Current	2805	23.8 (22.8–24.7)	646	13.2 (11.9–14.5)	2480	13.7 (13.0–14.3)
**Physical activity**						
Ideal	7497	54.4 (53.3–55.5)	2138	41.6 (39.8–43.4)	6710	36.3 (35.5–37.2)
Intermediate	2114	15.9 (15.1–16.7)	854	17.5 (16.1–19.0)	2496	13.9 (13.3–14.6)
Poor	4088	30.0 (28.6–30.7)	2280	40.8 (39.1–42.6)	8907	49.7 (48.8–50.6)
**BMI (kg/m**^**2**^**)**						
< 25.0	5644	42.2 (41.1–43.3)	2001	39.1 (37.3–40.9)	6609	37.2 (36.3–38.1)
25.0–29.9	4989	36.2 (35.1–37.3)	1928	36.3 (34.6–38.1)	6942	39.1 (38.2–40.0)
≥ 30.0	3066	21.6 (20.7–22.5)	1343	24.5 (23.0–26.0)	4562	23.7 (23.0–24.4)
**Healthy diet score**						
Ideal	438	3.5 (3.1–3.9)	206	4.5 (3.7–5.2)	335	1.9 (1.6–2.1)
Intermediate	12,644	92.4 (91.8–92.9)	3875	74.7 (73.1–76.3)	10,507	60.0 (59.1–60.9)
Poor	617	4.1 (3.7–4.5)	1191	20.8 (19.3–22.3)	7271	38.1 (37.2–39.0)
**TC (mg/dL)**						
< 200	8298	64.4 (63.3–65.4)	3493	70.1 (68.5–71.7)	15,317	86.3 (85.7–86.9)
200–239	3574	24.4 (23.5–25.3)	1260	22.2 (20.7–23.7)	2273	11.2 (10.7–11.8)
≥ 240	1827	11.2 (10.6–11.9)	519	7.8 (6.8–8.6)	523	2.4 (2.2–2.7)
**FPG (mg/dL)**						
< 100 (untreated)	10,912	82.3 (81.5–83.1)	3682	74.5 (730.–76.0)	13,014	75.6 (74.8–76.3)
100–125 or treated	1818	11.7 (11.1–12.4)	1041	18.2 (16.8–19.6)	3654	18.4 (17.7–19.1)
≥ 126	969	5.9 (5.4–6.4)	549	7.3 (6.4–8.1)	1445	6.0 (5.6–6.3)
**SBP/DBP (mmHg)**						
< 120/80 (untreated)	4064	34.0 (33.0–35.0)	1512	34.5 (32.7–36.2)	5784	36.7 (35.8–37.6)
120–139/80–89 or treated	5568	42.3 (41.2–43.4)	2138	42.7 (40.9–44.5)	7486	43.0 (42.0–44.0)
≥ 140/90	4067	23.7 (22.8–24.6)	1622	22.8 (21.4–24.2)	4843	20.4 (19.7–21.1)
**No. of ideal CVH metrics**						
0	15	0.17 (0.07–0.3)	6	0.15 (0.01–0.3)	12	0.08 (0.03–0.1)
1	595	4.8 (4.3–5.3)	213	4.8 (4.0–5.6)	712	4.3 (3.9–4.7)
2	2216	17.4 (16.5–18.2)	853	19.0 (17.6–20.5)	2917	18.0 (17.3–18.8)
3	3674	27.7 (26.7–28.7)	1267	25.7 (24.1–27.3)	4893	28.9 (27.9–29.6)
4	3818	27.2 (26.2–28.2)	1447	26.9 (25.2–28.5)	5360	28.7 (27.9–29.6)
5	2461	16.7 (15.9–17.5)	998	16.2 (14.9–17.5)	3240	15.7 (15.0–16.3)
6	848	5.5 (5.0–6.0)	459	6.7 (5.9–7.6)	893	3.4 (3.6–4.2)
7	72	0.5 (0.3–0.6)	29	0.5 (0.2–0.8)	86	0.4 (0.3–0.5)
Mean CVH metrics (95% CI)		4.7 (4.7–4.8)		4.9 (4.8–5.0)		5.0 (4.9–5.1)

Data from each survey is weighted for age, sex, area of residence, on the basis of the Iranian census population 2011.

STEPS, STEPwise approach to surveillance; CI, confidence interval; BMI, body mass index; TC, total cholesterol; FPG, fasting plasma glucose; SBP, systolic blood pressure; DBP, diastolic blood pressure; CVH metrics, cardiovascular health index. SI conversion factors: to convert total cholesterol values to mmol/L, multiply by 0.0259; to convert glucose values to mmol/L, multiply by 0.0555.

Tables 4 and 5 show the prevalence and trend of each CVH metrics in women and men, separately. We found that in each cycle, the prevalence of low physical activity and obesity but not overweight status were significantly higher in women than in men, while current smoking was more prevalent in men. The trends of all 7 CVH metrics during this period were generally similar to the trends among the total population (see above), except for overweight/obesity status, which remained constant in women and increased in men.

**Table 4:** Prevalence of cardiovascular health metrics among females in STEPS 2007, 2011, 2016.

	2007		2011		2016	
	No	Prevalence (95%CI)	No	Prevalence (95%CI)	No	Prevalence (95%CI)
**Smoking status**						
Never	6213	92.2 (91.4–92.9)	3137	96.4 (95.7–97.1)	9087	94.3 (93.8–94.8)
Former	72	1.0 (0.7–1.3)	24	0.4 (0.2–0.6)	323	2.6 (2.2–2.9)
Current	443	6.8 (6.1–7.5)	141	3.2 (2.5–3.8)	401	3.1 (2.7–3.5)
**Physical activity**						
Ideal	2869	41.0 (39.6–42.5)	1030	30.4 (28.3–32.4)	2619	24.8 (23.8–25.8)
Intermediate	1293	20.2 (19.0–21.5)	589	20.0 (18.1–21.8)	1451	15.0 (14.2–15.9)
Poor	2566	38.7 (37.2–40.2)	1683	49.6 (47.4–51.9)	5741	60.1 (58.9–61.3)
**BMI (kg/m**^**2**^**)**						
< 25.0	2120	32.2 (30.8–33.7)	1102	34.7 (32.5–37.0)	3060	32.6 (31.5–33.8)
25.0–29.9	2499	36.7 (35.2–38.2)	1176	34.8 (32.6–36.9)	3583	37.2 (36.0–38.4)
≥ 30.0	2109	31.0 (29.6–32.4)	1024	30.5 (28.4–32.6)	3168	30.2 (29.1–31.3)
**Health diet score**						
Ideal	224	3.8 (3.2–4.4)	133	4.6 (3.7–5.6)	188	1.9 (1.6–2.2)
Intermediate	6199	92.3 (91.5–93.1)	2444	76.0 (74.0–77.8)	5667	60.1 (59.0–61.3)
Poor	305	3.9 (3.4–4.4)	725	18.1 (17.7–21.2)	3956	38.0 (36.8–39.1)
**TC(mg/dL)**						
< 200	3755	60.5 (59.1–62.0)	2082	67.7 (65.6–67.7)	8119	85.7 (84.9–86.5)
200–239	1888	26.1 (24.8–27.4)	824	22.9 (21.1–24.8)	1341	11.6 (10.8–12.3)
≥ 240	1085	13.3 (12.3–14.3)	396	9.4 (8.1–10.6)	351	2.7 (2.4–3.06)
**FPG (mg/dL)**						
< 100 (untreated)	5296	82.1 (81.0–83.2)	2300	74.2 (72.2–76.1)	7036	76.4 (75.5–77.4)
100–125 or treated	899	11.5 (10.6–12.4)	645	18.3 (16.5–20.0)	1931	17.2 (16.3–18.1)
≥ 126	533	6.4 (5.7–7.13)	357	7.5 (6.4–8.6)	844	6.4 (5.9–6.8)
**SBP/DBP (mmHg)**						
< 120/80 (untreated)	2121	38.5 (36.9–40.0)	1026	38.6 (36.3–40.8)	3407	41.9 (40.7–43.1)
120–139/80–89 or treated	2519	37.0 (35.5–38.4)	1253	38.4 (36.2–40.6)	3715	37.6 (36.4–38.8)
≥ 140/90	2088	24.6 (23.3–25.8)	1023	23.0 (21.2–24.7)	2689	20.5 (19.7–21.4)
**No. of CVH metrics**						
0	7	0.3 (− 0.01–0.6)	4	0.1 (− 0.006–0.3)	5	0.06 (0.005–0.1)
1	284	4.0 (3.2–4.8)	136	5.2 (4.1–6.2)	343	3.9 (3.5–4.4)
2	999	15.7 (14.3–17.2)	504	18.4 (16.7–20.2)	1566	18.8 (17.9–19.7)
3	1747	24.9 (23.3–26.6)	768	26.1 (24.1–28.0)	2651	29.6 (28.6–30.7)
4	1929	25.3 (23.7–26.9)	923	27.5 (25.5–29.5)	3001	29.3 (28.2–30.3)
5	1326	21.6 (20.3–23.0)	649	15.4 (14.0–16.8)	1766	15.0 (14.1–15.6)
6	423	7.0 (6.2–7.7)	307	7.0 (6.0–8.0)	454	3.2 (2.9–3.6)
7	13	1.1 (0.9–1.18)	11	0.3 (0.05–0.5)	25	0.1 (0.08–0.2)
Mean CVH metrics (95% CI)		4.7 (4.7–4.8)		5.0 (4.9–5.1)		5.0 (5.0–5.1)

Data from each survey is weighted for age, sex, area of residence, on the basis of the Iranian census population 2011.

STEPS, STEPwise approach to surveillance; CI, confidence interval; BMI, body mass index; TC, total cholesterol; FPG, fasting plasma glucose; SBP, systolic blood pressure; DBP, diastolic blood pressure; ICVH, Ideal cardiovascular health metrics. SI conversion factors: To convert total cholesterol values to mmol/L, multiply by 0.0259; to convert glucose values to mmol/L, multiply by 0.0555.

**Table 5:** Prevalence of cardiovascular health metrics in among males in STEPS 2007, 2011, 2016.

	2007		2011		2016	
	No	Prevalence (95%CI)	No	Prevalence (95%CI)	No	Prevalence (95%CI)
**Smoking status**						
Never	3609	48.8 (47.3–40.3)	1280	65.1 (62.2–68.0)	5110	64.5 (63.2–65.8)
Former	1000	14.2 (13.0–15.3)	185	7.7 (6.2–9.3)	1113	11.0 (10.2–11.8)
Current	2362	37.0 (35.6–38.5)	505	27.1 (24.4–29.8)	2079	24.5 (23.3–25.7)
**Physical activity**						
Ideal	4628	64.9 (63.4–66.4)	1108	57.2 (54.2–60.2)	4091	48.1 (46.7–49.5)
Intermediate	821	12.5 (11.4–13.6)	265	14.2 (12.0–16.4)	1045	12.8 (11.9–13.7)
Poor	1522	22.6 (21.2–23.9)	597	28.6 (25.9–31.3)	3166	39.1 (37.7–40.4)
**BMI (kg/m**^**2**^**)**						
< 25.0	3524	50.(48.4–51.6)	899	45.2 (42.2–48.2)	3549	41.8 (40.5–43.2)
25.0–29.9	2490	35.8 (34.3–37.3)	752	38.5 (35.5–41.5)	3359	41.1 (39.7–42.4)
≥ 30	957	14.2 (13.0–15.3)	319	16.2 (14.0–18.4)	1394	17.1 (16.0–18.1)
**Health diet score**						
Ideal	214	3.2 (2.7–3.8)	214	4.3 (3.0–5.5)	147	1.9 (1.5–2.2)
Intermediate	6445	92.5 (91.7–93.2)	6445	73.0 (70.4–75.7)	4840	59.9 (58.5–61.2)
Poor	312	4.3 (3.7–4.9)	312	22.7 (20.2–25.2)	3315	38.2 (36.9–39.6)
**Cholesterol (mg/dL)**						
< 200	4543	67.3 (65.9–68.8)	1411	73.4 (70.8–76.1)	7198	87.0 (86.0–87.9)
200–239	1686	23.0 (21.8–24.3)	436	21.2 (18.8–23.7)	932	10.9 (10.0–11.7)
≥ 240	742	9.6 (8.7–10.5)	123	5.3 (4.0–6.6)	172	2.2 (1.7–2.6)
**FPG (mg/dL)**						
< 100 (untreated)	5616	82.5 (81.4–83.6)	1382	75.0 (72.4–77.4)	5978	74.7 (73.6–76.0)
100–125 or treated	919	11.9 (10.9–12.9)	396	18.1 (15.8–20.4)	1723	19.7 (18.6–20.7)
≥ 126	436	5.6 (4.9–6.3)	192	6.9 (5.6–8.2)	601	5.6 (5.1–6.2)
**SBP/DBP (mmHg)**						
< 120/80 (untreated)	1943	30.4 (28.9–31.8)	486	28.8 (26.0–31.6)	2377	31.3 (30.–32.6)
120–139/80–89 or treated	3049	46.6 (45.0–48.1)	885	48.6 (45.6–51.7)	3771	48.3 (47.0–49.7)
≥ 140/90	1979	23.1 (21.8–24.3)	599	22.6 (21.2–24.9)	2154	20.3 (19.2–21.3)
**No. of CVH metrics**						
0	8	0.1 (0.02–0.2)	2	0.1 (− 0.05–0.3)	7	0.1 (0.02–0.17)
1	311	4.0 (3.1–4.8)	77	5.0 (3.7–6.3)	369	4.8 (4.2–5.3)
2	1217	15.7 (14.2–17.2)	349	21.0 (18.6–23.4)	1351	17.6 (16.6–18.6)
3	1927	27.0 (25.0–29.0)	499	25.9 (23.3–28.4)	2242	28.3 (27.2–29.5)
4	1889	27.0 (25.0–29.0)	524	25.5 (23.1–28.0)	2359	27.9 (26.7–29.1)
5	1135	18.3 (16.6–20.0)	349	16.2 (14.2–18.3)	1474	16.1 (15.2–17.1)
6	425	7.1 (6.0–8.3)	152	5.5 (4.4–6.6)	439	4.4 (3.9–4.9)
7	59	0.8 (0.5–1.2)	18	0.7 (0.2–1.3)	61	0.6 (0.4–0.8)
Mean CVH metrics (95% CI)		4.7 (4.6–4.8)		4.8 (4.7–4.9)		5.0 (4.9–5.1)

Data from each survey is weighted for age, sex, area of residence, on the basis of Iranian census population 2011.

STEPS, STEPwise approach to surveillance; BMI, body mass index; TC, total cholesterol; FPG, fasting plasma glucose; SBP, systolic blood pressure; DBP, diastolic blood pressure; ICVH, Ideal cardiovascular health metrics. SI conversion factors: to convert total cholesterol values to mmol/L, multiply by 0.0259; to convert glucose values to mmol/L, multiply by 0.0555.

**Figure 2:**
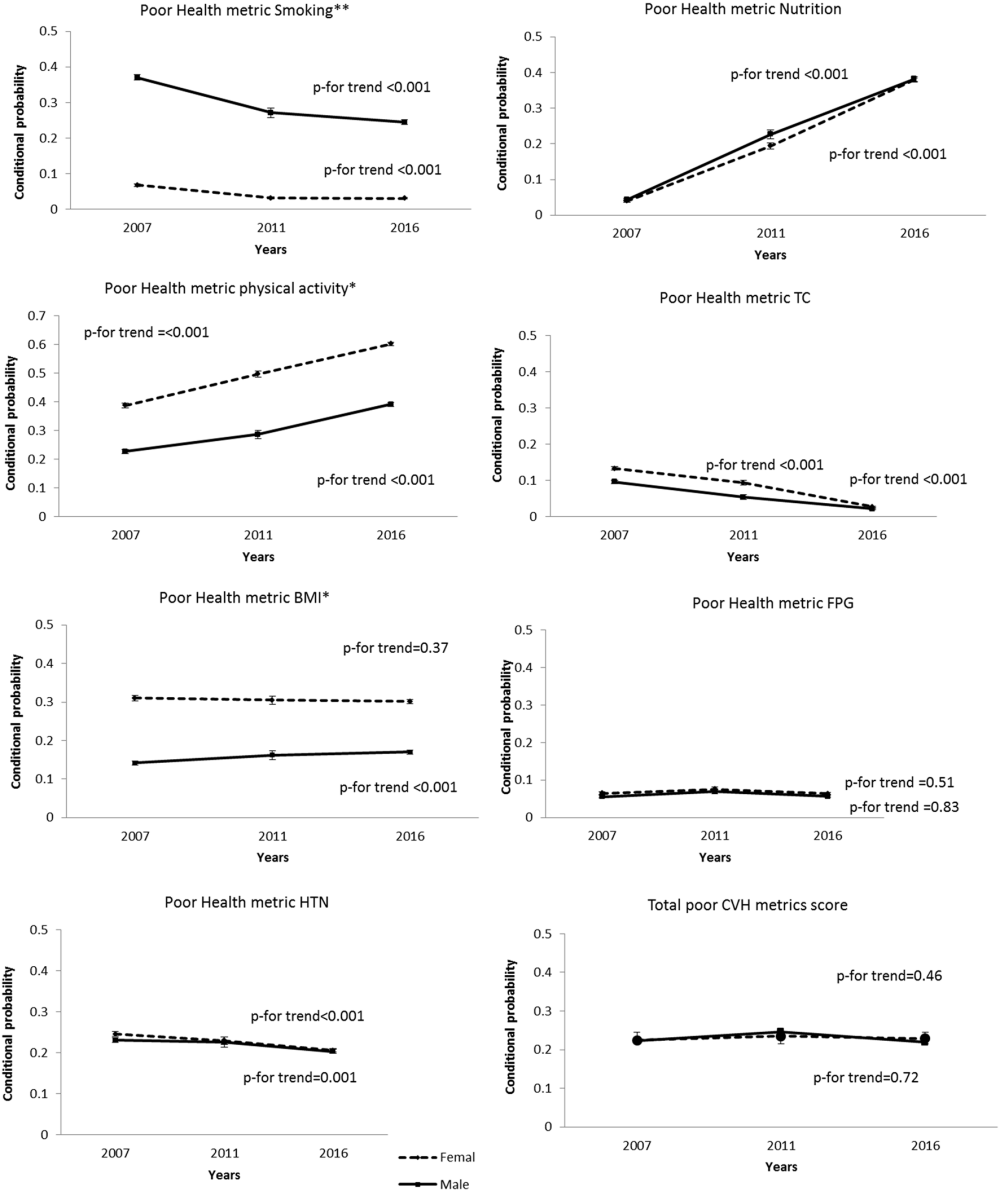
Conditional probability and trend of seven poor health metrics compared to ideal and intermediate categories as a reference among men and women. **0.0001 < *p* value ≤ 0.001; *0.001 < *p* value ≤ 0.05. TC, total cholesterol; BMI, body mass index; FPG, fasting plasma glucose; HTN, hypertension; CVH, cardiovascular health.

Figure 2 shows the trend of each poor CVH metrics compared with the composite of the ideal and intermediate categories [as references] among men and women during 2007–2016. Accordingly, although poor smoking status showed a noticeable downward trend during the study period in both sexes, the issue was more prominent in women. In both genders, we found a significant downward trend for physical activity, with this issue being more accentuated for men. Moreover, despite a high prevalence of obesity among women compared with men during the study, obesity remained constant in women but showed an increasing trend in men. We did not find significant differences in the trends of poor healthy diet score, TC ≥ 240 mg/dl, FPG ≥ 126 mg/dl, and HTN status between genders.

## Discussion

In the current study, we examined the trends of poor, intermediate, and ideal levels of CVH metrics during 3 consecutive national surveys from 2007 to 2016. Throughout these surveys, the prevalence of BP level ≥ 140/90 mmHg, TC ≥ 240 mg/dl, and current smoking status decreased in both genders. However, overweight and obesity status increased among men, and physical activity of less than 600 MET mins/wk and modified poor diet score increased in both genders. Moreover, the prevalence of prediabetes (but not diabetes state) increased significantly in both genders. Unfortunately, only about 6% and 3.8% of the total population met 6 or more ideal CVH metrics in 2007 and 2016, respectively.

The low prevalence of ideal CVH metrics is a global health problem, notably in low-and middle-income countries^8^. To the best of our knowledge, 4 studies conducted in developing countries (India, Iran, Bosnia/Herzegovina, and Ecuador), exhibited a low prevalence of ≥ 6 ideal CVH metrics (0.3–4%)^5^. Similarly, in the current study, although this prevalence reached about 7.2% in 2011, it again decreased to < 4% in 2016. The consistently low trends of ideal CVH metrics were also confirmed in national studies conducted in the US (i.e. < 10%)^4^, Canada (< 8.8%)^7^, and Korea (< 14%)^6^. Moreover, the intermediate categories of CVH metrics (i.e. having 3, 4, and 5 ideal CVH metrics) had the highest prevalence in both genders in this study (≈ 70%). Comparable values in NHANES and KNHANES were 64.4–66.3% and 66.7–71%, respectively^4,6^.

The trend of current smoking status was shown to decline from 23.8 to 13.7%. The trend analysis of previous studies in the US and non-US population also reflect the improvement of smoking status^5^. This reduction rate was more pronounced among women, a finding similar to that observed in the KNHANES and Canadian study^6,7^. Moreover, it is important to note that Iranian women are more likely to underreport smoking behaviors, given the social shame of smoking^17^. Unfortunately, in 2016, about 1 out of 4 Iranian men were current smokers. Importantly, more than 19% of the population attributable fraction (PAF) of premature CVD among men was attributed to current smoking^18^. This risk might be minimized by effective programs such as raising taxes on cigarettes as well as the development of a workplace smoking regulation and smoking restriction at the national level.

The favorable trend of poor total cholesterol level in our study (11.2% in 2007 to 2.4% in 2016) was significantly better than a comparable change in the US (20.6% in 1988–1994 to 14.2% in 2005–2010)^4^ and Korea (5.4% in 2005 to 7.6% in 2009)^6^. The decreasing trend in lipid levels in our data registry could hardly be explained by lifestyle changes (i.e. increasing physical activity) since it was shown that low physical activity increased from 30.0 to 49.7% during this period, or by the use of lipid-lowering medications (only 5.6% in 2016). Despite this, due to extensive media advertising against the consumption of saturated fatty acids, it was shown that over 30% of Iranian families now consume less hydrogenated oil than they used to in the past^19^, which could explain the favorable lipid trend in the Iranian population during our surveys.

Poor diet scores stemming from low consumption of fruits, vegetables, and fish demonstrated a significant increase from 4.1% in 2007 to 38.1% in 2016 in our study. The average daily consumption of fruits and vegetables among the Iranian adult population in 2007 (2.58 servings per day) was reported to be lower than the average value recommended by WHO (5 or more servings per day)^20^. Our results were in line with nutritional status worldwide, with a healthy diet score being reported to be the poorest CVH metrics in the previous US and non-US studies^5^. According to Global Burden of Disease results, the consumption of whole-grain in the MENA region was about one-third of the global average^8^; hence, this downward shift in nutritional status would be more pronounced if this item had been included in our study. From 2007 to 2016, rising living costs and rising inflation that might be attributable to the country's long years of sanctions reduced people's access to healthy diets that contain enough vegetables and fruits. This issue, along with the numerous preoccupations of people to earn enough income, has led to not allocating enough time to prepare healthy diets, in other words, the food pattern has increasingly changed to a Western diet incorporating caloric dense and sucrose-enriched drinks^21^. Also, the occurrence of natural disasters such as floods, earthquakes, and droughts frequently during these years along with political instability in the region, especially in neighboring countries, has become an important reason for the growing decline in healthy nutrition in the people^22^.

Recent reports have shown that the mean physical activity level in the MENA region was approximately 1000 MET mins/wk less than the average global value for both genders^8^. This low physical activity level might be explained by rapid urbanization, industrialization, and a sedentary lifestyle due to occupational and transport facility changes in the region^21^. On the other hand, in contrast to the results of NHANES and KNHANES, the trend of low physical activity increased from 2007 to 2016, with a significantly higher slope in men, although women had significantly higher values of physical inactivity across all three surveys. The higher prevalence of physical inactivity among women may be linked to their role as caregiver to another family member in the house, their lower-income, and cultural issues that limit the participation of women in some sports^23^. To promote physical activity, public health agencies should reinforce appropriate public health education and organize strategic intervention programs among adults in Iran, such as the development of public sports facilities.

Regarding pre-hypertension and hypertension status, the favorable trend observed in 2005–2011^19^, continued until 2016. Despite this, in 2016, 3 out of 5 adults had pre-hypertension/hypertension. Accordingly, among the participants of the Tehran Lipid and Glucose Study (TLGS), the highest PAF for CVD among potential risk factors was attributed to hypertension, reaching 21.6%^24^. Unfortunately, the prevalence of pre-hypertension remained constant at about 43% during this period. Importantly, the presence of prehypertension was shown to be significantly associated with CVD and mortality^25,26^. The slight favorable trend of hypertension in contrast to low physical activity, poor diet status, and high salt intake^27^ might be justified by other contributing factors such as the increase in the use of antihypertensive drugs. The high prevalence of intermediate and poor BP levels among Iranian residents requires immediate preventive programs, consisting of encouraging physical activity, reducing salt intake, as well as increasing the consumption of fruits, vegetables, and a potassium-rich diet.

The prevalence of BMI ≥ 25 kg/m^2^ and FPG ≥ 100 mg/dl (including drug-treated patients) in the study population increased from about 58 to 63%, and 18 to 24%, respectively from 2007 to 2016. Based on the study in 2011, it was estimated that the age-standardized prevalence of IFG and type 2 diabetes among the Iranian population aged ≥ 20 years was 14.4% and 11.3%, respectively^28^. Data from the 9th edition of the International Diabetes Federation reported that 463 million people suffer from diabetes around the world. Without effective national advocacy, this will be expected to increase by 51%, reaching 700 million in 2045; most of this increase is expected to arise from the MENA region^29^. The significant increase in abnormalities in glucose metabolism could be related to an aging population, unhealthy lifestyle, and physical inactivity due to a growing urban population. In our study, the prevalence of obesity in each survey was higher among women than in men, similar to other countries in the MENA region^30^. The high prevalence of obesity among Iranian women might be attributable to the high prevalence of low physical activity as discussed above; moreover, the high parity rate among women was addressed as an underlying factor of obesity in this gender^31^. Among Iranians, the PAF of BMI ≥ 25 kg/m^2^ for type 2 diabetes among women and men was 34.6% and 27.1%, respectively^28^. The TLGS demonstrated that among modifiable risk factors, the PAF of diabetes for CVD and all-cause mortality were about 14% and 24%, respectively, while the PAF of CVD attributed to being overweight or obese was about 24%^24^. Hence, the significant increase in the prevalence of being overweight/obese and impaired glucose intolerance could negate the favorable trend of hypercholesterolemia, smoking, and hypertension. To address these paradoxical trends, implementing public health interventions that target lifestyle change by educating people regarding the advantages of a healthy diet and increasing physical activity is seriously advised; the impact of which was shown for up to 6 years in reducing metabolic syndrome among Tehranian residents.

Implementing multicomponent community-based prevention strategies in the form of NGOs and charitable organizations, health marketing strategies to raise public awareness about health behaviors, using community health workers (Behvarz) especially in deprived rural areas, applying smartphone preventive program to improve the quality of health care, adopting general practitioners and other health workers to assign community members to a link worker, and finally preventive, diagnostic, and therapeutic services may improve cardiovascular health status. Major gains will likely achieve from public health strategies targeting incorrect diet, physical inactivity, and preventing or stopping cigarette smoking^32^. As a successful example, in a community-based study in Tehran, it was shown that family–school and community based educational sessions resulted in about 20% decrease incidence of metabolic syndrome during 6 years follow up^33^.

### Strengths and limitations

To the best of our knowledge, this is one of the few studies examining the trends of CVH metrics (alongside the US, Canadian, and Korean studies). Moreover, the fact that this study measured 7 CVH metrics over 10 years and examined trends in each gender is another strong point. However, our study had some limitations. First, we used a subsample of the STEPS population; namely those with complete data on CVH metrics, which might diminish the exact assessment of CVH metrics trends in the Iranian population. Second, the sampling method performed in 2016 differed slightly from 2007 and 2011; however, all samples taken in the three surveys were representative of the Iranian population. Third, self-reporting of physical activity, cigarette smoking, and dietary records in our surveys could cause recall bias and imprecise estimation of cardiovascular health status. Fourth, only 2 components of a healthy diet were used for assessment of nutritional status and our modified diet score may not reflect the complete effect of diet on cardiovascular health status.

## Conclusion

The prevalence of ideal CVH metrics remained very low during 2007–2016 and in the last survey, less than 4% of Iranian adults met the criteria of 6 or more ideal CVH metrics. Although the levels of smoking, TC, and BP had improved slightly, the unfavorable trends of physical activity, unhealthy diet, obesity, and ideal FPG need immediate attention at a public health level using a multi-component strategy.

## Data Availability

Data are available from the corresponding author on reasonable request.

## Abbreviations

BP: Blood pressure
BMI: Body mass index
CVH metrics: Cardiovascular health metrics
CVD: Cardiovascular disease
CI: Confidence interval
FPG: Fasting plasma glucose
GPAQ: Global physical activity questionnaire
KNHANES: Korean National Health and Nutrition Examination Survey
METs: Metabolic equivalent tasks
NHANES: National Health and Nutrition Examination Survey
STEPS: STEPwise approaches to surveillance
TC: Total cholesterol
TLGS: Tehran Lipid and Glucose Study

## References

[1] Fang J, Yang Q, Hong Y, Loustalot F (2012). Status of cardiovascular health among adult Americans in the 50 states and the district of Columbia, 2009. J. Am. Heart Assoc..

[2] Shay CM (2012). Status of cardiovascular health in US adults: prevalence estimates from the National Health and Nutrition Examination Surveys (NHANES) 2003–2008. Circulation.

[3] Lloyd-Jones DM (2010). Defining and setting national goals for cardiovascular health promotion and disease reduction: the American Heart Association’s strategic Impact Goal through 2020 and beyond. Circulation.

[4] Yang Q (2012). Trends in cardiovascular health metrics and associations with all-cause and CVD mortality among US adults. JAMA.

[5] Younus, A. *et al.* In: *Mayo Clinic Proceedings.* 649–670 (Elsevier, Amsterdam).

[6] Lee H-J, Suh B, Yoo T-G, Lee H, Shin DW (2013). Trends in cardiovascular health metrics among Korean adults. Korean J. Family Med..

[7] Maclagan LC (2014). The **CANHEART** health index: a tool for monitoring the cardiovascular health of the Canadian population. CMAJ.

[8] Azizi F (2019). Metabolic health in the Middle East and north Africa. Lancet Diabetes Endocrinol.

[9] Malekzadeh, R. Health in Iran Burden of Diseases and risk factors. *Islamic Republic of Iran Ministry of Health and Medical Education, Deputy of Research and Technology*. http://ir-de.iust.ac.ir/irde-storage/2018/03/103-MoHME_Malekzadeh.pdf (10 accessed December 2019) (2018).

[10] Moghaddam MM (2014). Distribution of ideal cardiovascular health in a community-based cohort of Middle East population. Ann. Saudi Med..

[11] Esteghamati A (2009). Third national surveillance of risk factors of non-communicable diseases (SuRFNCD-2007) in Iran: methods and results on prevalence of diabetes, hypertension, obesity, central obesity, and dyslipidemia. BMC Public Health.

[12] Hajipour MJ (2017). Protocol design for large-scale cross-sectional studies of surveillance of risk factors of non-communicable diseases in Iran: STEPs 2016. Arch. Iran. Med..

[13] Delavari, A., Alikhani, S. & Alaedini, F. A national profile of non-communicable disease risk factors in the IR of Iran. Center for Disease Control, Tehran*. *www.ncdinfobase.ir/docs.asp (2005).

[14] Riley L (2016). The World Health Organization STEPwise approach to noncommunicable disease risk-factor surveillance: methods, challenges, and opportunities. Am. J. Public Health.

[15] Armstrong T, Bull F (2006). Development of the world health organization global physical activity questionnaire (GPAQ). J. Public Health..

[16] Folsom AR (2011). Community prevalence of ideal cardiovascular health, by the American Heart Association definition, and relationship with cardiovascular disease incidence. J. Am. Coll. Cardiol..

[17] Parizadeh D (2019). Sex-specific initiation rates of tobacco smoking and its determinants among adults from a Middle Eastern population: a cohort study. Int. J. Public Health.

[18] Eslami A (2017). Sex-specific incidence rates and risk factors of premature cardiovascular disease. A long term follow up of the Tehran Lipid and Glucose Study. Int. J. Cardiol..

[19] Kheirandish M, Asgari S, Lotfaliany M, Bozorgmanesh M, Saadat N, Tohidi M, Azizi F, Hadaegh F (2014). Secular trends in serum lipid levels of a Middle Eastern adult population; 10 years follow up in Tehran lipid and glucose study. Lipids Health Dis..

[20] Esteghamati A (2012). Patterns of fruit and vegetable consumption among Iranian adults: a SuRFNCD-2007 study. Br. J. Nutr..

[21] Esteghamati A (2010). Secular trends of obesity in Iran between 1999 and 2007: national surveys of risk factors of non-communicable diseases. Metab. Syndr. Relat. Disord..

[22] Programme, W. H. F. Food and nutrition security in Iran. A Summary Report, WFP Iran Country Office (2016).

[23] Esteghamati A (2011). Physical activity in Iran: results of the third national surveillance of risk factors of non-communicable diseases (SuRFNCD-2007). J. Phys. Act. Health.

[24] Sardarinia M (2016). Risk factors for incidence of cardiovascular diseases and all-cause mortality in a middle eastern population over a decade follow-up: Tehran lipid and glucose study. PLoS ONE.

[25] Guo X (2013). Association between pre-hypertension and cardiovascular outcomes: a systematic review and meta-analysis of prospective studies. Curr. Hypertens. Rep..

[26] Huang Y (2014). Association of all-cause and cardiovascular mortality with prehypertension: a meta-analysis. Am. Heart J..

[27] Rezaei S (2018). Salt intake among Iranian population: the first national report on salt intake in Iran. J. Hypertens..

[28] Esteghamati A (2014). Trends in the prevalence of diabetes and impaired fasting glucose in association with obesity in Iran: 2005–2011. Diabetes Res. Clin. Pract..

[29] International Diabetes Federation. IDF diabetes atlas ninth edition 2019.

[30] Abarca-Gómez L (2017). Worldwide trends in body-mass index, underweight, overweight, and obesity from 1975 to 2016: a pooled analysis of 2416 population-based measurement studies in 128.9 million children, adolescents, and adults. Lancet.

[31] Brooks R, Maklakov A (2010). Sex differences in obesity associated with total fertility rate. PLoS ONE.

[32] Alizadeh G, Gholipour K, Khosravi MF, Khodayari-Zarnaq R (2020). Preventive community-based strategies of cardiovascular diseases in Iran: a multi-method study. Soc. Work Public Health..

[33] Lotfaliany M (2020). Long-term effectiveness of a lifestyle intervention on the prevention of type 2 diabetes in a middle-income country. Sci. Rep..

